# CTLA-4 polymorphisms associate with breast cancer susceptibility in Asians: a meta-analysis

**DOI:** 10.7717/peerj.2815

**Published:** 2017-01-10

**Authors:** Zhiming Dai, Tian Tian, Meng Wang, Xinghan Liu, Shuai Lin, Pengtao Yang, Kang Liu, Yi Zheng, Peng Xu, Meng Liu, Xuewen Yang, Zhijun Dai

**Affiliations:** 1 Department of Oncology, Second Affiliated Hospital of Xi’an Jiaotong University, Xi’an, China; 2 Department of Anesthesia, Second Affiliated Hospital of Xi’an Jiaotong University, Xi’an, China

**Keywords:** CTLA-4, Polymorphism, Breast cancer, Susceptibility, Meta-analysis

## Abstract

Previous studies have investigated the association between cytotoxic T-lymphocyte antigen-4 (*CTLA-4*) polymorphisms and breast cancer susceptibility, but the results remained inconsistent. Therefore, we evaluated the relationship between four common *CTLA-4* polymorphisms and breast cancer risk by a meta-analysis, aiming to derive a comprehensive and precise conclusion. We searched EMBASE, Pubmed, Web of Science, CNKI, and Wanfang databases until July 18th, 2016. Finally, ten eligible studies involving 4,544 breast cancer patients and 4,515 cancer-free controls were included; all these studies were from Asia. Odds ratio (OR) and 95% confidence interval (CI) were used to evaluate the breast cancer risk in five genetic models. The results indicated that the *CTLA-4* +49A>G (rs231775) polymorphism had a significant association with decreased breast cancer risk in allelic, homozygous, dominant and recessive models. Also, the +6230G>A (rs3087243) polymorphism reduced breast cancer risk especially in the Chinese population under homozygous and recessive models. In contrast, the −1661A>G (rs4553808) polymorphism increased breast cancer risk in allelic, heterozygous and dominant models, whereas −1722 T>C (rs733618) did not relate to breast cancer risk. In conclusion, *CTLA-4* polymorphisms significantly associate with breast cancer susceptibility in Asian populations, and different gene loci may have different effects on breast cancer development. Further large-scale studies including multi-racial populations are required to confirm our findings.

## Introduction

Breast cancer has been the most common type of cancer and the main cause of cancer death among women in the world, which was estimated to have 1.7 million new cases in 2012 (Torre et al., 2015). Breast cancer is an extremely heterogeneous disease in the clinic and the potential molecular mechanism of carcinogenesis has not been clearly understood so far. In recent years, inherited factors were identified to play a critical role in the development of breast cancer (Reeves et al., 2012).

Researches on the field of tumour immunology found that the immune system can influence tumour occurence during the period of elimination, equilibrium and escape (Dunn, Old & Schreiber, 2004). Cytotoxic T-lymphocyte antigen-4 (CTLA-4), which was also designated as CD152, expressed mainly on activated T cells. As an immunosuppressive cytokine, it can inhibit T-lymphocyte proliferation and activation (Sun et al., 2008). Numerous researches have demonstrated that blockage of CTLA-4 function can improve antitumor immunity (Leach, Krummel & Allison, 1996; Ribas et al., 2004; Vandenborre et al., 1999). This indicates CTLA-4 may exert positive effects on carcinogenesis. The human *CTLA-4* gene, which is located in human chromosome 2q33, is one of the most important genes involved in immune responses to a variety of antigens (Walunas et al., 2011). *CTLA-4* gene comprises four exons and has several important polymorphisms in the entire region, including the +49G>A (rs231775) in exon 1 (Donner et al., 1997), the +6230G>A (rs3087243) in 3^′^-untranslated region (Hughes, 2006), the −1661A>G (rs4553808) and −1722 T>C (rs733618) in the promoter region (Johnson et al., 2001), which are the most commonly studied single nucleotide polymorphisms (SNPs). These SNPs are important because they can alter the host immune response by affecting the transcription of *CTLA-4* gene, the expression of CTLA-4 protein, and the interaction of CTLA-4 and CD80 ligand (Anjos et al., 2002; Sun et al., 2008; Wang et al., 2002).

Numerous investigations have demonstrated that *CTLA-4* genetic polymorphisms may have association with human breast cancer susceptibility (Erfani et al., 2006; Ghaderi et al., 2004; Li et al., 2012; Li et al., 2008; Minhas et al., 2014; Sun et al., 2008; Wang et al., 2007; Zhifu et al., 2015; Kong, 2010). The results showed that some of the polymorphisms such as rs733618 and rs4553808 may increase the breast cancer risk while other polymorphisms such as rs231775 and rs3087243 may reduce the risk of breast cancer. Considering that a single study does not have enough power to detect the overall effects, we conducted a meta-analysis which is a statistical analysis of the data from some collection of studies in order to synthesize the results to obtain a more reliable evaluation of the relationship between the four common SNPs in *CTLA-4* gene and breast cancer susceptibility.

## Materials and Methods

Our meta-analysis was conducted according to the Preferred Reporting Items for Systematic Reviews and Meta-Analyses (PRISMA) guidelines (Moher et al., 2010).

### Search strategy

We searched the databases of EMBASE, PubMed, Web of Science, Wanfang, as well as Chinese National Knowledge Infrastructure (CNKI) to identify all the relevant articles up to July 18th, 2016. Keywords for search were: “CTLA-4 or Cytotoxic T-lymphocyte antigen 4 or CD152,” “polymorphism or variation or SNP or rs231775 or rs308724 or rs4553808 or rs733618,” and “breast cancer.” The eligible article must be published in English or Chinese. References in retrieved articles were also searched manually.

### Criteria for selection

All studies selected for further meta-analysis must conform to the included criteria: (1) case-control study conducted in human and investigated the association of SNPs in *CTLA-4* with breast cancer susceptibility; (2) All the breast cancer patients were diagnosed by pathology or histology; (3) detailed data of the allele and genotype distributions are available; (4) the controls were cancer-free individuals. In addition, articles meet the following criteria were excluded: (1) articles that were reviews, conference abstracts, or repeat publications; (2) study design were based on family; (3) studies with no control groups. The quality of each included study was assessed by the Newcastle-Ottawa Scale for case-control studies (Wells et al., 2014).

### Data extraction

According to the selection criteria, two authors (Zhiming Dai and Tian Tian) reviewed the literature independently and extracted the raw data and information from each eligible study including: first author, publication year, country of origin, racial ancestry, source of control, genotype method, total number of case and control, allele frequency and genotype distribution in case and control, and *P* value of HWE in control. Any discrepancy was discussed between authors and refereed by Zhijun Dai to reach a consensus.

### Statistical analysis

For each study, ORs and 95% CIs were computed to estimate the breast cancer risk associated with *CTLA-4* polymorphisms. Pooled ORs were calculated under the following genetic models: allele comparison of B *vs.* A, homozygote of BB *vs.* AA, heterozygote of AB *vs.* AA, dominant model of (BB + AB) *vs.* AA and recessive model of BB *vs.* (AA + AB). Heterogeneity among studies were estimated by *I*^2^ test and chi^2^-based Q statistic and significance was considered at *I*^2^>50% (Higgins & Thompson, 2002). We adopted the random-effects model to analyze the combined ORs if *I*^2^ value was greater than 50%. Otherwise, a fixed-effects model should be exerted (Petitti, 2001). We carried out subgroup analysis to estimate the specific effects of ethnicity and source of control. We also conducted a sensitivity analysis to assess the consistency and stability of our meta-analysis by omitting individual study in turn. Additionally, Begg’s funnel plot and Egger’s test were used to assess publication bias, and significance was identified as *P* < 0.05 (Begg & Mazumdar, 1994; Egger et al., 1997). All the statistical analyses were implemented with the Review Manager (Version 5.3; Cochrane Collaboration, London, UK) and STATA software (Version 12.0; Stata Corp, College Station, TX, USA).

## Results

### Characteristics of included studies

**Figure 1:**
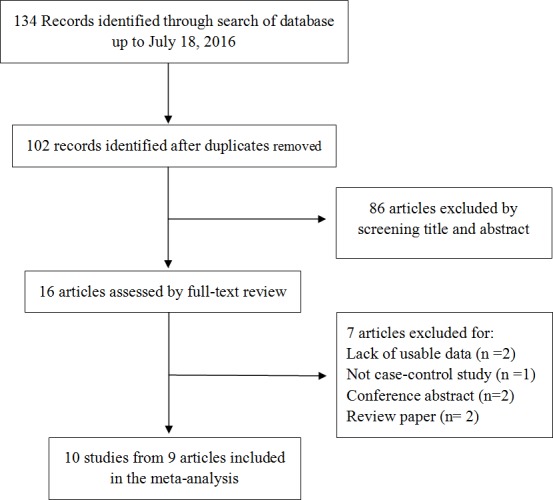
Flow chart of the studies selection.

Finally, nine articles comprising 10 studies investigating *CTLA-4* +49A>G (rs231775) and/or +6230G>A (rs3087243) and/or −1722 T>C (rs733618) and/or −1661A/G (rs4553808) polymorphisms were identified for further analysis (Fig. 1). Table 1 presented the characteristics of selected studies. Of the 10 studies, seven were from China, two were from Iran, and one was from India. Additionally, seven studies were based on population and three based on hospitals. Moreover, genotype distributions in the control group of each included studies complied with Hardy-Weinberg equilibriums (HWE) (*P* > 0.05) except for one study for one SNP (Erfani et al., 2006). The detailed data of the allele frequency and genotype distribution as well as HWE from each study were shown in Table 2.

**Table 1:** Characteristics of the studies included in the meta-analysis.

First author	Year	Country	Ethnicity	Genotyping medthod	Source of control	Total sample size (case/control)	SNP
Yu	2015	China	Chinese	PCR-RFLP	PB	376/366	1;2;3;4
Minhas	2014	India	Indian	PCR-RFLP	PB	250/250	1
Li D	2012	China	Chinese	PCR-RFLP	PB	581/566	1;2;3;4
Kong	2010	China	Chinese	PCR-RFLP	HB	315/322	4
Sun	2008a	China	Chinese	PCR-RFLP	PB	1060/1070	1
Sun	2008b	China	Chinese	PCR-RFLP	PB	1037/1070	1
Li H	2008	China	Chinese	PCR-RFLP	HB	328/327	2;3
Wang	2007	China	Chinese	PCR-RFLP	PB	117/148	1;2;4
Erfani	2006	Iran	Iranian	PCR-CTPP	PB	283/245	3;4
Ghaderi	2004	Iran	Iranian	PCR-RFLP	HB	197/151	1

**Notes.**

PCR polymerase chain reaction RFLP restriction fragment length polymorphism CTPP confronting two pairs primers PB population based HB hospital based SNP single-nucleotide polymorphism

SNP No.1, +49A>G (rs231775); 2, +6230G>A (rs3087243); 3, −1722T>C (rs733618); 4, −1661A>G (rs4553808).

**Table 2:** Genotype distributions and allele frequencies of *CTLA-4* polymorphisms in cases and controls.

Study	Genotype (N)								Allele frequency (N)				*P* of HWE
	Case				Control				Case		Control		
	Total	AA	AB	BB	Total	AA	AB	BB	A	B	A	B	
+49A>G (rs231775)													
Zhifu et al. (2015)	376	174	175	27	366	174	157	35	523	229	505	227	0.96
Minhas et al. (2014)	250	111	113	26	250	105	121	24	335	165	331	169	0.20
Li et al. (2012)	576	49	281	246	553	54	243	256	379	773	351	755	0.74
Sun et al. (2008)	1,060	101	485	474	1,070	65	446	559	660	1,406	576	1,564	0.15
Sun et al. (2008)	1,037	100	455	482	1,070	73	451	546	655	1,419	597	1,543	0.12
Wang et al. (2007)	117	48	59	10	148	55	70	23	155	79	180	116	0.93
Ghaderi et al. (2004)	197	84	104	9	151	60	72	19	272	122	192	110	0.72
+6230G>A (rs3087243)													
Zhifu et al. (2015)	376	257	110	9	366	252	103	11	624	128	607	125	0.90
Li et al. (2012)	581	361	197	23	566	361	182	23	919	243	904	228	0.99
Li et al. (2008)	328	32	124	172	327	20	114	193	188	468	154	500	0.57
Wang et al. (2007)	117	24	47	46	148	18	56	74	95	139	92	204	0.16
−1722T>C (rs733618)													
Zhifu et al. (2015)	376	123	186	67	366	137	166	63	432	320	440	292	0.30
Li et al. (2012)	574	184	276	114	551	207	256	88	644	504	670	432	0.55
Li et al. (2008)	328	125	163	40	327	111	168	48	413	243	390	264	0.22
Erfani et al. (2006)	282	225	54	3	245	204	41	0	504	60	449	41	0.15
−1661A>G (rs4553808)													
Zhifu et al. (2015)	376	273	91	12	366	281	78	7	637	115	640	92	0.56
Li et al. (2012)	574	405	153	16	551	425	115	11	963	185	965	137	0.33
Kong (2010)	315	204	105	6	322	241	76	5	513	117	558	86	0.72
Wang et al. (2007)	109	62	45	2	148	111	35	2	169	49	257	39	0.68
Erfani et al. (2006)	282	211	65	6	238	184	43	11	487	77	411	65	0.001

**Notes.**

A the major allele B the minor allele HWE Hardy-Weinberg equilibrium

### Meta-analysis results

**Figure 2:**
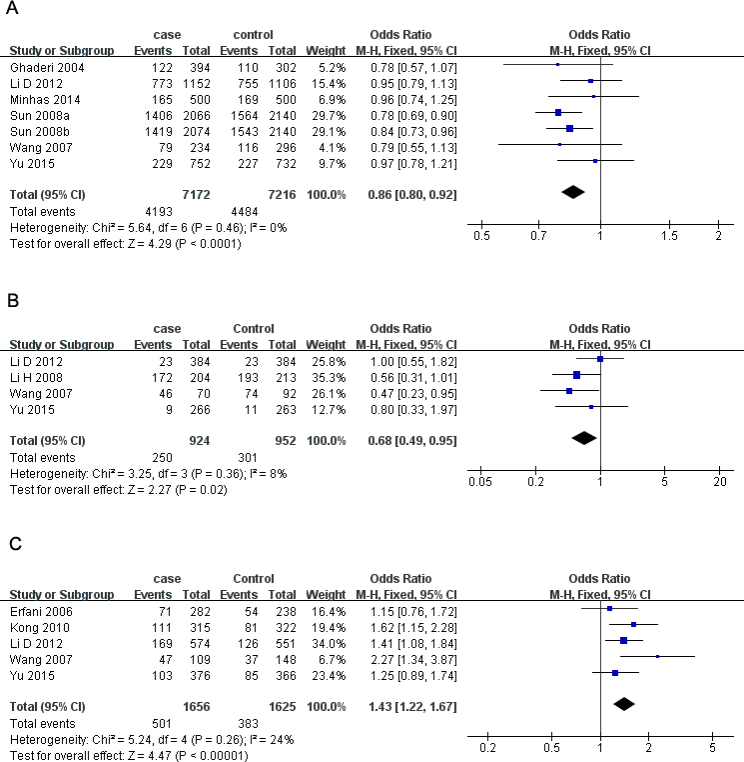
Forest plots of *CTLA-4* polymorphisms and breast cancer risk. (A) rs231775 under G * vs.* A; (B) rs3087243) under AA * vs.* GG; (C) rs4553808 under AG + GG * vs.* AA. The squares and horizontal lines correspond to the study-specific OR and 95% CI. The area of the squares reflects the weight (inverse of the variance). The diamond represents the summary OR and 95% CI.

Seven studies containing 3,613 cases and 3,608 controls focused on breast cancer risk with *CTLA-4* rs231775 polymorphism. As presented in Table 3, significantly decreased risk was observed in the overall population in all the models except heterozygote (G *vs.* A: *OR* = 0.86, 95% CI [0.80–0.92], *P* = 0.000, Fig. 2A; GG *vs.* AA: *OR* = 0.68, 95% CI [0.57–0.81], *P* = 0.000; GG *vs.* AA + AG: *OR* = 0.79, 95% CI [0.71–0.87], *P* = 0.000; AG + GG *vs.* AA: *OR* = 0.85, 95% CI [0.74–0.97], *P* = 0.02;). In subgroup analyses, rs231775 was also found to significantly reduce the breast cancer risk in Chinese and subgroup based on population under allelic, homozygous and recessive models.

**Table 3:** Meta-analysis results of *CTLA-4* polymorphisms and BC risk.

SNP	B *vs.* A		BB *vs.* AA		AB *vs.* AA		BB *vs.* AA + AB		AB + BB *vs.* AA	
	OR (95%CI)	*P*	OR (95%CI)	*P*	OR (95%CI)	*P*	OR (95%CI)	*P*	OR (95%CI)	*P*
+49A>G (rs231775)										
Overall	0.86 (0.80–0.92)	**0.000**	0.68 (0.57–0.81)	**0.000**	0.92 (0.80–1.06)	0.23	0.79 (0.71–0.87)	**0.000**	0.85 (0.74–0.97)	**0.02**
Chinese	0.85 (0.79–0.92)	**0.000**	0.68 (0.56–0.82)	**0.000**	0.92 (0.73–1.16)	0.49	0.79 (0.71–0.88)	**0.000**	0.84 (0.65–1.08)	0.17
PB	0.86 (0.80–0.93)	**0.000**	0.70 (0.59–0.84)	**0.000**	0.91 (0.78–1.05)	0.19	0.80 (0.72–0.89)	**0.000**	0.85 (0.69–1.04)	0.12
+6230G>A (rs3087243)										
Chinese	0.87 (0.71–1.07)	0.20	0.68 (0.49–0.95)	**0.02**	0.99 (0.83–1.19)	0.94	0.77 (0.61–0.97)	**0.02**	0.87 (0.65–1.17)	0.36
PB	0.91 (0.71–1.17)	0.48	0.75 (0.50–1.12)	0.15	1.03 (0.85–1.25)	0.76	0.77 (0.54–1.09)	0.14	1.00 (0.82–1.20)	0.99
−1722T>C (rs733618)										
Overall	1.09 (0.93–1.29)	0.29	1.15 (0.79–1.68)	0.47	1.13 (0.96–1.32)	0.15	1.11 (0.90–1.37)	0.32	1.14 (0.98–1.33)	0.09
Chinese	1.07 (0.88–1.29)	0.51	1.12 (0.77–1.63)	0.55	1.12 (0.94–1.33)	0.22	1.10 (0.89–1.35)	0.39	1.11 (0.86–1.43)	0.43
PB	1.19 (1.05–1.34)	**0.007**	1.37 (1.05–1.78)	**0.02**	1.22 (1.02–1.47)	**0.03**	1.21 (0.96–1.54)	0.11	1.26 (1.06–1.50)	**0.01**
−1661A>G (rs4553808)										
Overall	1.34 (1.16–1.53)	**0.000**	1.22 (0.78–1.92)	0.38	1.45 (1.23–1.70)	**0.000**	1.12 (0.72–1.76)	0.61	1.43 (1.22–1.67)	**0.000**
Chinese	1.41 (1.21–1.63)	**0.000**	1.59 (0.95–2.67)	0.08	1.47 (1.24–1.75)	**0.000**	1.45 (0.86–2.43)	0.16	1.48 (1.25–1.75)	**0.000**
PB	1.30 (1.11–1.52)	**0.001**	1.19 (0.73–1.94)	0.48	1.40 (1.17–1.68)	**0.000**	1.11 (0.68–1.80)	0.68	1.38 (1.16–1.64)	**0.000**
HWE	1.41 (1.21–1.63)	**0.000**	1.59 (0.95–2.67)	0.08	1.47 (1.24–1.75)	**0.000**	1.45 (0.86–2.43)	0.16	1.48 (1.25–1.75)	**0.000**

**Notes.**

A the major allele B the minor allele CI confidence interval OR odds ratio PB population based HB hospital based SNP single-nucleotide polymorphism HWE subgroup excluding the study departing from HWE

There were four studies all of which were from China with 1,402 cases and 1,407 controls investigating the relationship between and breast cancer susceptibility and *CTLA-4* rs3087243 polymorphism. The results presented a significantly lower breast cancer risk in homozygous and recessive genetic models in Chinese women (AA *vs.* GG: *OR* = 0.68, 95% CI [0.49–0.95], *P* = 0.02, Fig. 2B; AA *vs.* GG + GA: *OR* = 0.77, 95% CI [0.61–0.97], *P* = 0.02).

For *CTLA-4* rs733618 polymorphism, we aseessed 4 studies containing 1,560 cases and 1,489 controls. Overall, our analysis did not suggest any association between rs733618 and breast cancer susceptibility. However, when stratifying by source of control, rs733618 was observed to increase breast cancer risk based on population in three genetic models (C *vs.* T: *OR* = 1.19, 95% CI [1.05–1.34], *P* = 0.007; CC *vs.* TT: *OR* = 1.37, 95% CI [1.05–1.78], *P* = 0.02; CT *vs.* TT: *OR* = 1.22, 95% CI [1.02–1.47], *P* = 0.03).

Five studies involving 1,656 cases and 1,625 controls investigated the breast cancer risk with* CTLA-4* rs4553808 polymorphism. We observed a higher risk in overall analysis under three models (G *vs.* A: *OR* = 1.34, 95% CI [1.16–1.53], *P* = 0.000; AG *vs.* AA: *OR* = 1.45, 95% CI [1.23–1.70], *P* = 0.000; AG + GG *vs.* AA: *OR* = 1.43, 95% CI [1.22–1.67], *P* = 0.000, Fig. 2C). The results were similar in Chinese subgroup. When stratifying by source of control, rs4553808 was also noted to increase breast cancer risk in allelic and heterozygous models based on population.

### Heterogeneity analysis and sensitivity analysis

As presented in Table 4, no obvious heterogeneity was detected for the four *CTLA-4* polymorphisms in most of the genetic models. For the few in which existed significant heterogeneity (*I*^2^ > 50%), random-effects model was applied.

**Table 4:** Heterogeneity-analysis results of *CTLA-4* polymorphisms and BC risk.

SNP	B *vs.* A			BB *vs.* AA			AB *vs.* AA			BB *vs.* AA + AB			AB + BB *vs.* AA		
	*I*^2^	*P*	EM	*I*^2^	*P*	EM	*I*^2^	*P*	EM	*I*^2^	*P*	EM	*I*^2^	*P*	EM
+49A>G (rs231775)															
Overall	0%	0.46	F	45%	0.09	F	29%	0.21	F	27%	0.22	F	41%	0.12	F
Chinese	12%	0.34	F	40%	0.15	F	51%	0.09	R	0%	0.59	F	60%	0.04	R
PB	6%	0.38	F	39%	0.14	F	39%	0.15	F	0%	0.56	F	51%	0.07	R
+6230G>A (rs3087243)															
Chinese	58%	0.07	R	8%	0.36	F	0%	0.38	F	0%	0.78	F	53%	0.09	R
PB	60%	0.08	R	24%	0.27	F	16%	0.31	F	0%	0.58	F	46%	0.16	F
−1722T>C (rs733618)															
Overall	52%	0.10	R	51%	0.10	R	7%	0.36	F	35%	0.22	F	39%	0.18	F
Chinese	64%	0.06	R	59%	0.09	R	37%	0.21	F	31%	0.23	F	57%	0.10	R
PB	0%	0.74	F	0%	0.45	F	0%	0.99	F	0%	0.37	F	0%	0.98	F
−1661A>G (rs4553808)															
Overall	27%	0.24	F	9%	0.35	F	14%	0.32	F	6%	0.37	F	24%	0.26	F
Chinese	0%	0.48	F	0%	0.99	F	32%	0.22	F	0%	0.98	F	24%	0.27	F
PB	39%	0.18	F	31%	0.23	F	27%	0.25	F	30%	0.24	F	34%	0.21	F
HWE	0%	0.48	F	0%	0.99	F	32%	0.22	F	0%	0.98	F	24%	0.27	F

**Notes.**

EM Effects model F fixed effects model R random effects model PB population based HB hospital based SNP single-nucleotide polymorphism HWE subgroup excluding the study departing from HWE

**Figure 3:**
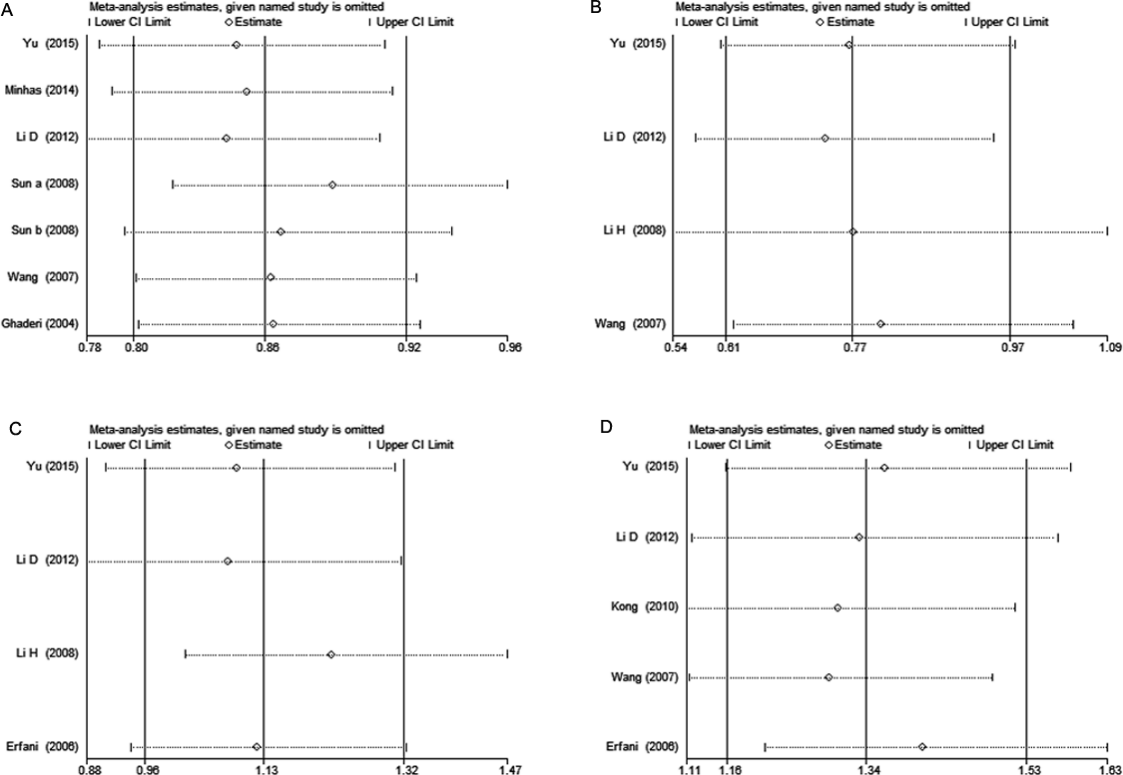
Sensitivity analysis of * CTLA-4* polymorphisms and breast cancer risk. (A) rs231775 under G *vs.* A; (B) rs3087243 under AA *vs.* GG + GA; (C) rs733618 under TC *vs.* TT; (D) rs4553808 under G *vs.* A. Each point represents the pooled OR after omitting single study in left column. The two ends of the dotted lines represent the 95% CI.

Each study was sequentially removed to assess the impact of single study on the combined ORs. The result showed that the omission of any study didn’t alter the overall estimations substantially, indicating that our meta-analysis results were robust (Fig. 3).

### Publication bias

**Figure 4:**
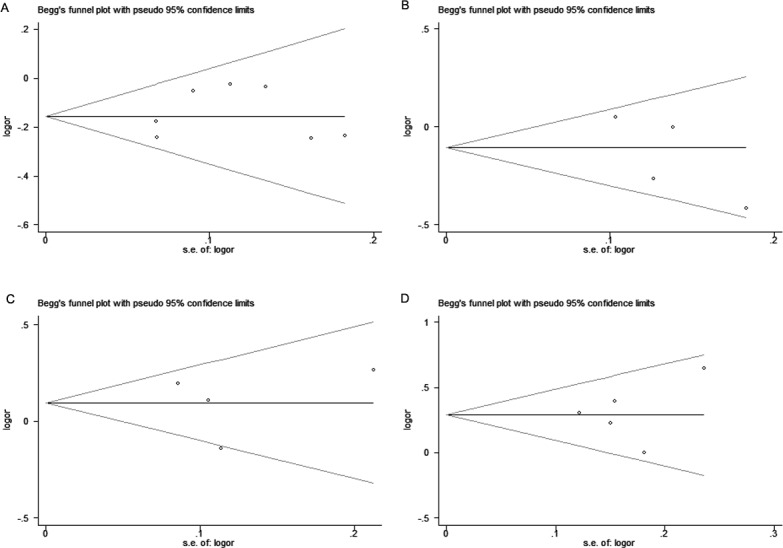
Begg’s funnel plots of publication bias for the association of *CTLA-4* polymorphisms and breast cancer risk. (A) rs231775, (B) rs3087243, (C) rs733618, (D) rs4553808 under the allelic model. Each point represents a single study for the indicated association.

We implemened Begg’s funnel plot and Egger’s test to asesess the publication bias. As shown in Fig. 4, funnel plot failed to display obvious asymmetry. The Egger’s test result didn’t reveal any publication bias for the four SNPs in *CTLA-4* gene and breast cancer risk either (Table 5, *P* > 0.05).

## Discussion

It was reported that mutation in human *CTLA-4* gene resulted in quantitative reduction of CTLA-4 expression and led to a severe immunoregulatory disorder (Kuehn et al., 2014). Several investigations have suggested that particular *CTLA-4* gene polymorphisms are linked to cancer development or progression (Erfani et al., 2006; Tang et al., 2014; Wang et al., 2007). However, the results from those studies remained conflicting. In one previous study, the author found that rs733618 and rs4553808 polymorphisms in *CTLA-4* increased the breast cancer risk whereas rs231775 and rs3087243 polymorphisms did not have significant associations with breast cancer risk (Li et al., 2012). However, in other studies, rs3087243 and rs231775 polymorphisms were found to reduce the risk of breast cancer while rs733618 did not associated with breast cancer risk (Sun et al., 2008; Wang et al., 2007). Since CTLA-4 is important in carcinogenesis and a single study does not have enough statistical power to detect the effects, we carried out this meta-analysis which synthesized the results of the included studies with a statistical analysis of the data from these studies to draw a more reliable conclusion about the association between *CTLA-4* SNPs and breast cancer susceptibility.

In the present meta-analysis, we identified that *CTLA-4* rs231775 had an association with breast cancer susceptibility. We observed a significantly decreased risk in both overall and subgroup analysis in different genetic models. Some previous meta-analyses have also involved the relationship between rs231775 polymorphism and several tumor sites including breast cancer (Gao et al., 2014; Geng et al., 2014; Wang et al., 2015; Zhang et al., 2011). The results suggested the A allele of rs231775 may contribute to breast cancer susceptibility. Our result confirmed that the A allele of *CTLA-4* rs231775 polymorphism has more possibility to increase breast cancer risk than G allele. Nevertheless, our study differs from theirs because we specifically focused on breast cancer and our meta-analysis included more studies than theirs. Therefore, our results are more reliable.

**Table 5:** Egger’s test result of *CTLA-4* polymorphisms and BC risk based on allele frequency.

SNP	Coefficient	SE	*t*	*P*	95% CI
rs231775	0.71	1.13	0.63	0.557	−2.19–3.61
rs3087243	−5.19	3.20	−1.62	0.246	−18.94–0.56
rs733618	−0.03	3.17	−0.01	0.993	−13.67–13.61
rs4553808	1.26	2.82	0.45	0.686	−7.70–10.21

**Notes.**

SNP single-nucleotide polymorphism 95% CI 95% confidence interval SE standard error

*CTLA-4* rs3087243 polymorphism was found to decrease breast cancer risk under homozygous and recessive genetic models in Chinese. Our results implied that individuals carry AA genotype are less susceptible to breast cancer than those carry GG or (GG + GA) genotypes. Previous studies also found that rs3087243 was associated with breast cancer susceptibility as a subgroup of several cancer sites (Yan et al., 2013; Zhao, Duan & Gu, 2014). The results were similar with our research, but our our meta-analysis included one more study and have more statistical power.

We didn’t find any relationship between *CTLA-4* rs733618 and breast cancer risk in the overall analysis under any genetic model. However, there was a higher risk in the population-based group under all the genetic models except recessive model. Considering that we selected only four eligible studies and most of them were small-size sample (<500), these results need to be taken with caution. One previous study found a positive signal of rs733618 polymorphism with breast cancer (Li et al., 2012) while other two studies showed negative signal (Erfani et al., 2006; Tang et al., 2014). So further researches with larger sample size should be designed and implemented to validate or refute these conclusions.

In contrast, *CTLA-4* rs4553808 polymorphism was realated to an increased breast cancer risk in both overall and Chinese population in allelic, heterozygous and dominant models. This suggested that *CTLA-4* −1661G allele is more likely to be a risk factor of breast cancer than A allele. Previous meta-analysis also found rs4553808 may increase cancer risk especially for breast cancer (Geng et al., 2014; Yan et al., 2013; Zhao, Duan & Gu, 2014). But these studies investigated the association of this single SNP with various types of cancer while our study specifically focused on breast cancer and investigated several SNPs. Notably, for this SNP, the *P*-value of HWE in control of one study was less than 0.05 (Erfani et al., 2006), suggesting that the study population was not representative of a broad population. Nevertheless, we decided to keep this study because deleting it did not affect the pooled ORs significantly.

Several limitations of our research should be noticed. Firstly, the sample size in this meta-analysis was relatively small, especially for rs3087243, rs733618 and rs4553808 polymorphisms. Secondly, our results need to be interpreted with caution since we did not find any studies from Europe, Africa or America and most of the included studies were from China. Therefore, more studies with large population and more ethnic groups are needed to provide sufficient statistical power. Thirdly, other factors such as environmental variants, age, and living habit are generally considered to contribute to cancer susceptibility. Lacking data of these factors for adjustment may impact the estimation of breast cancer risk. Lastly, bias may still exist because we failed to find any studies of other races and we did not have access to gray literature.

## Conclusion

In summary, our meta-analysis suggests that rs231775, rs3087243 and rs4553808 polymorphisms in human *CTLA-4* gene significantly associated with breast cancer susceptibility in Asians, particularly in the Chinese population. In consideration of the limitations of our work, further large-scale studies including multi-racial populations are required to confirm our findings.

##  Supplemental Information

10.7717/peerj.2815/supp-1 Table S1 PRISMA checklist

10.7717/peerj.2815/supp-2 Supplemental Information 1 Excluded articles and the reasons for exclusion of each article

## Additional Information and Declarations

### Data Availability

The following information was supplied regarding data availability: The raw data is included in Tables 1 and 2.
