# Supplementary material for: CTLA-4 polymorphisms associate with breast cancer susceptibility in Asians: a meta-analysis
Source: PeerJ. 2017 Jan 10;5:e2815. doi: 10.7717/peerj.2815 (PMC5228500; doi:10.7717/peerj.2815)
Supplement: Supplemental Information 1 [file peerj-05-2815-s002.doc]

1. Yeganeh F, Pezeshki A. M, Talei, A. Dehaghani, A. S, Doroudchi M, Farjadian S, KalantariT, Ghaderi A. CTLA-4 gene polymorphism in Southern Iranian women with breast cancer. *International Journal of Cancer.* 186-186

Reason for exclusion: conference abstract

1. Dmytruk I, Makukh H, Tyrkus M. 2013. The cytotoxic T-cell lymphocyte antigen 4 gene allele polymorphisms among cancer patients from Ukraine. *Biochimica Clinica.* 37: S166.

Reason for exclusion: conference abstract

1. Minhas S, Bhalla S, Saxena R, Verma I. C, Aggarwal S. CTLA-4+49 G/A polymorphism is associated with lymph node metastasis: A study in 500 North Indian breast cancer patients. *European Journal of Cancer.* 51: S272-S273

Reason for exclusion: not case-control study

1. De Bruin, M. A, Ford, J. M, Kurian, A. W. Genetic polymorphisms as predictors of breast cancer risk. *Current Breast Cancer Reports.* 4:232-239.

Reason for exclusion: review

1. Sun T, Hu ZB, Shen HB, Lin DX.2009. Genetic polymorphisms in cytotoxic T-lymphocyte antigen 4 and cancer: the dialectical nature of subtle human immune dysregulation. *Cancer research.*15: 6011-6014.

Reason for exclusion: review

1. Weifeng Tang, Hao Qiu, Heping Jiang, Bin Sun, Lixin Wang, Jun Yin, Haiyong Gu. 2014. Lack of association between cytotoxic T-lymphocyte antigen 4 (CTLA-4) -1722T/C (rs733618) polymorphism and cancer risk: from a case-control study to a meta-analysis. *PLoS ONE*. 9:e94039.

Reason for exclusion: no usable data

1. [Ben Zhang](http://www.sciencedirect.com/science/article/pii/S1470204511700766), [Alicia Beeghly-Fadiel](http://www.sciencedirect.com/science/article/pii/S1470204511700766), [Jirong Long](http://www.sciencedirect.com/science/article/pii/S1470204511700766), [Wei Zheng](http://www.sciencedirect.com/science/article/pii/S1470204511700766).2011. Genetic variants associated with breast-cancer risk: comprehensive research synopsis, meta-analysis, and epidemiological evidence. *The Lancet Oncology*. 12: 477–488.

Reason for exclusion: no usable data
